# Supplementary figures and images for: Mutations in PIK3C2A cause syndromic short stature, skeletal abnormalities, and cataracts associated with ciliary dysfunction
Source: PLoS Genet. 2019 Apr 29;15(4):e1008088. doi: 10.1371/journal.pgen.1008088 (PMC6508738; doi:10.1371/journal.pgen.1008088)

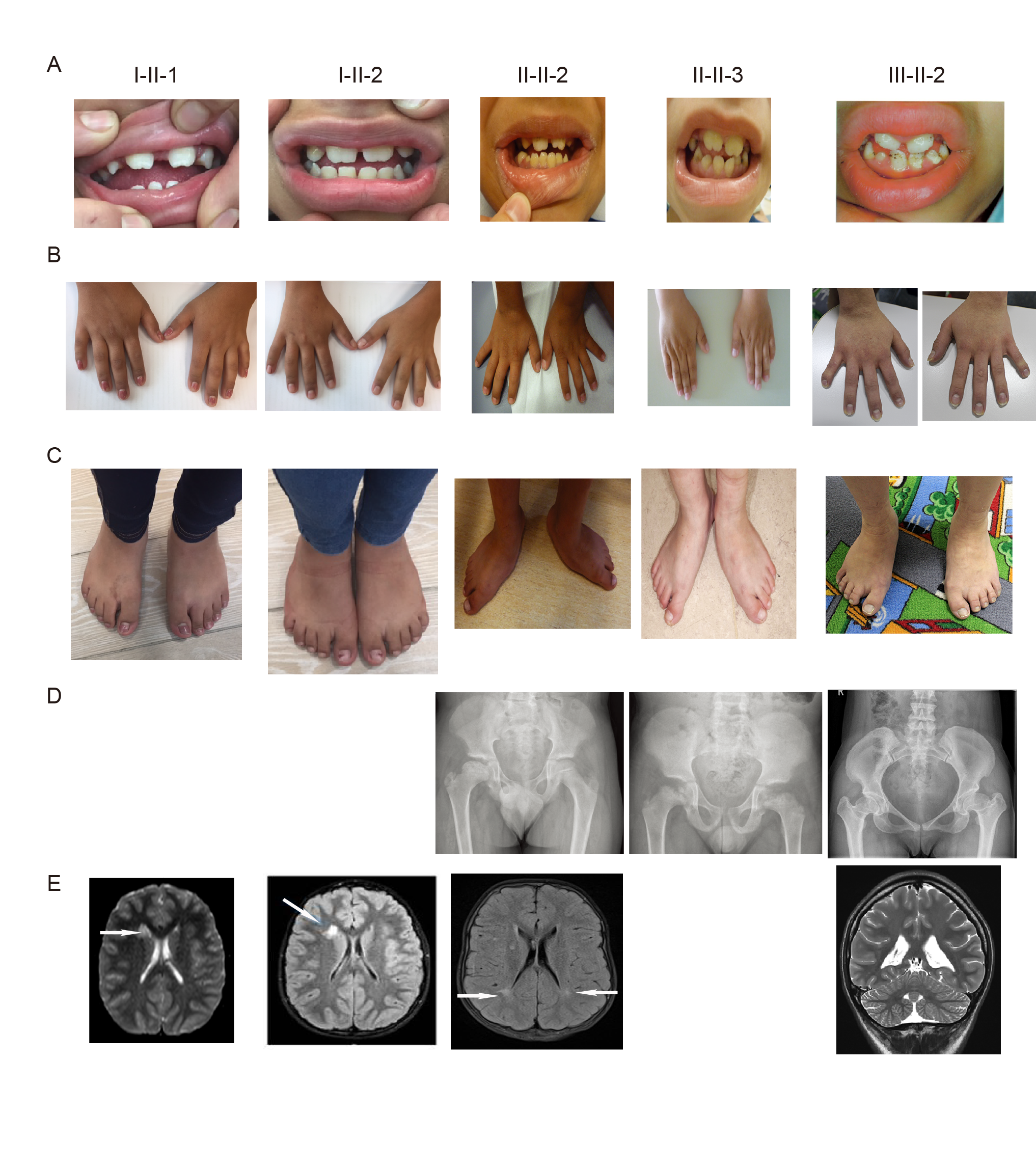

Supplement: S1 Fig — Photographic images of (A) teeth, (B) hands, and (C) feet are shown from the five individuals with PIK3C2A deficiency. (D) X-Ray images of the pelvis and (E) MRI images of the brain are shown when available. White arrows in the MRI images indicate regions of altered signal intensity. (TIF) [file pgen.1008088.s001.tif]

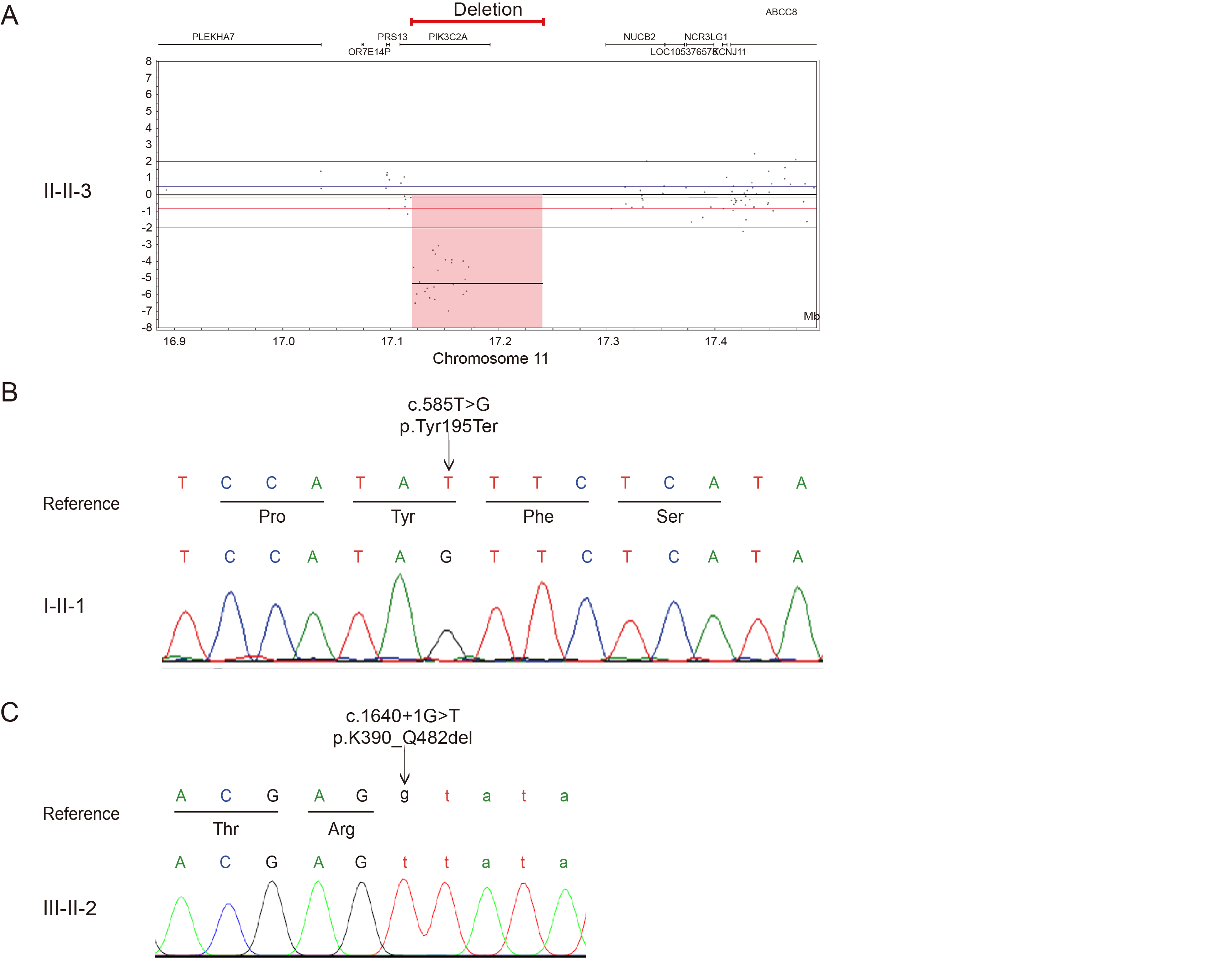

Supplement: S2 Fig — (A) CNV analysis confirmed a homozygous deletion encompassing exons 1–24 out of 32 total exons of PIK3C2A, indicated with the red line (B) Sanger sequencing confirmed homozygosity for the PIK3C2A c.585T variant in Family I. (C) Sanger sequencing confirmed homozygosity for the PIK3C2A c.1640+1 G>T variant in Family III. (TIF) [file pgen.1008088.s002.tif]

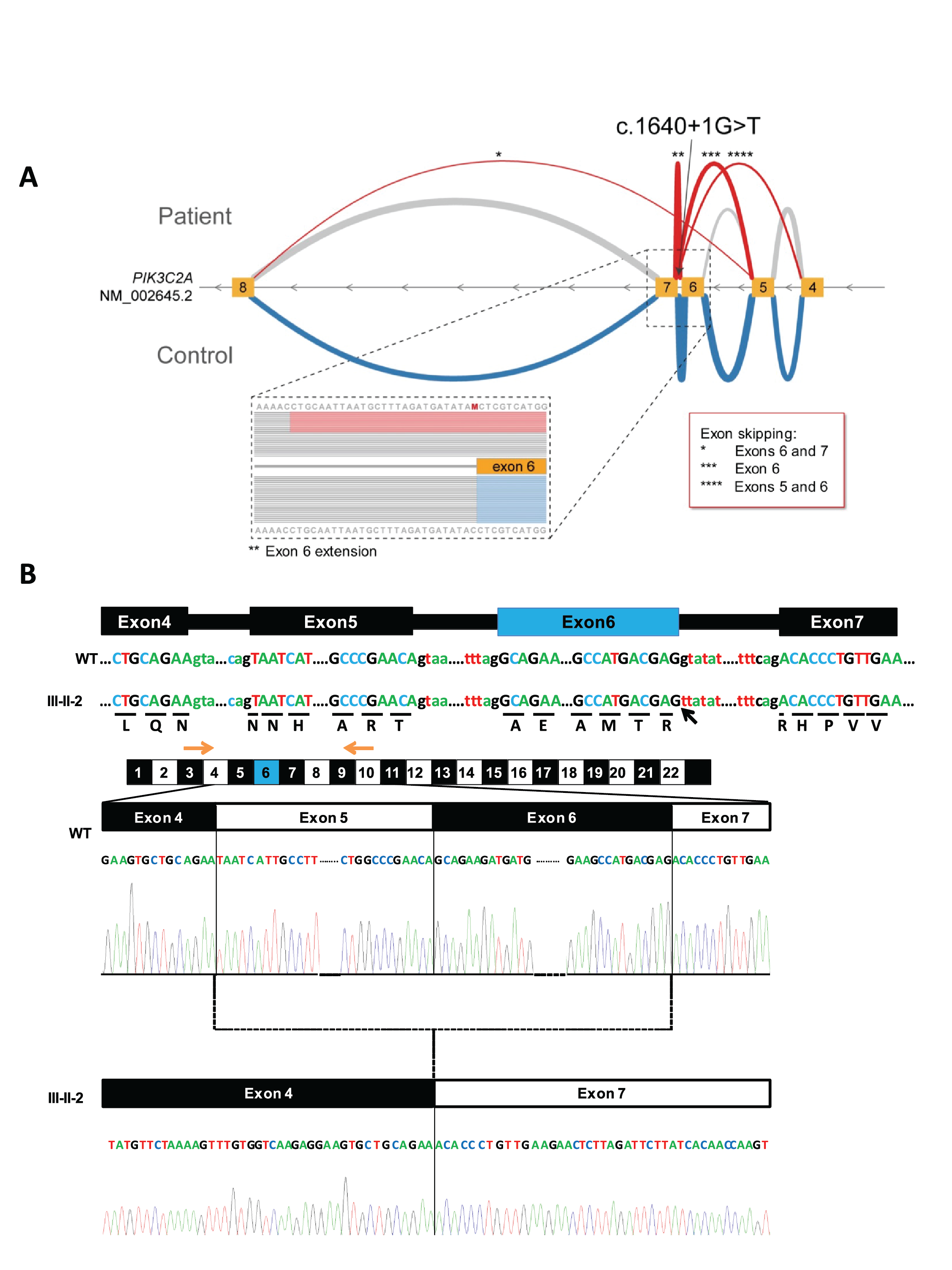

Supplement: S3 Fig — (A) Deep sequencing of RT-PCR products revealed 4 alternative transcripts in lymphocytes (red lines) compared to control samples (blue lines): *: r.1561_1704del; p.Ala521_Glu568del, **: r. 1640_1641ins1640+1_1640+27; p. Arg547SerinsTyrIleIle*, ***: r.1561_1640del; p.Ala521Thrfs*4, ****: r.1449_1640del; p.Asn483_Arg547delinsLys. Exon/intron structure of PIK3C2A (exons: yellow boxes) with transcripts of the patient of family III compared to controls. (B) Example of sequenced RT-PCR products from cDNA of fibroblasts from wild-type control and the patient of family III using primers located in exons 3 and 10. Skipping of exons 5 and 6 is the result of the mutation. Positions of primers are indicated by orange arrows and position of the splice site mutation is indicated by a black arrow. (TIF) [file pgen.1008088.s003.tif]

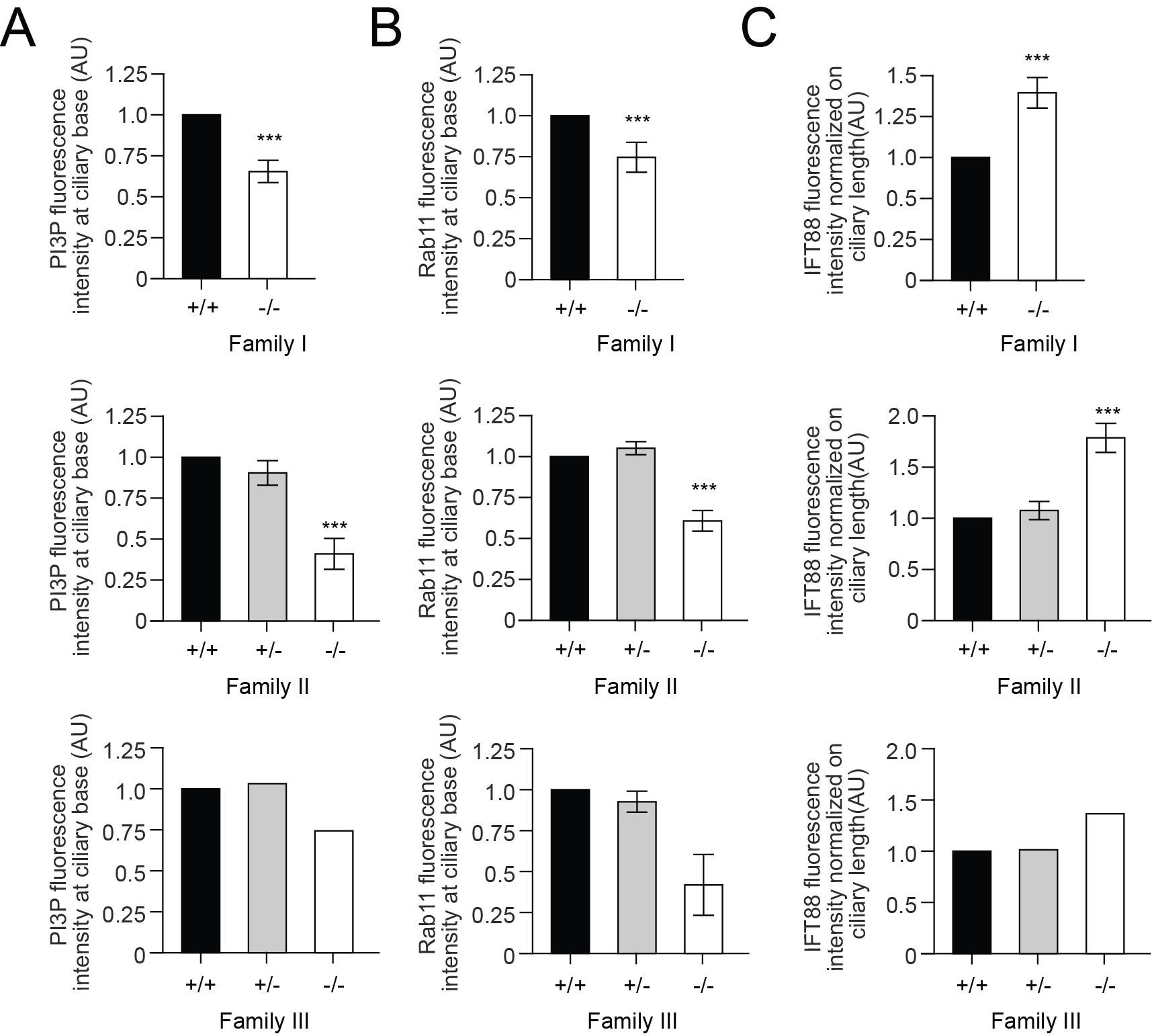

Supplement: S4 Fig — Quantification of fluorescence intensity for (A) PI(3)P at the ciliary base, (B) RAB11 at the ciliary base, and (C) ciliary IFT88. Data is shown separately for each individual family as indicated. (TIF) [file pgen.1008088.s004.tif]
